# Yields, Phenolic Profiles and Antioxidant Activities of *Ziziphus jujube* Mill. in Response to Different Fertilization Treatments

**DOI:** 10.3390/molecules181012029

**Published:** 2013-09-27

**Authors:** Chun-Sen Wu, Qing-Han Gao, Roger Keith Kjelgren, Xu-Dan Guo, Min Wang

**Affiliations:** 1 College of Food Science and Engineering, Northwest A & F University, Yangling 712100, Shaanxi, China; E-Mail: wuchunsen@126.com; 2 School of Public Health, Ningxia Medical University, Yinchuan 750004, Ningxia, China; E-Mail: gaoqinghan85@126.com; 3 Department of Plants, Soils, and Climate, College of Agriculture, Utah State University, Logan, UT 84322, USA; E-Mail: roger.kjelgren@usu.edu; 4 Chinese Cereals and Oils Association, Beijing 100037, China; E-Mail: guoxudan123@126.com

**Keywords:** jujube, organic fertilizer, inorganic fertilizer, phenolics, antioxidant activity

## Abstract

Increasing demand for more jujube (*Ziziphus jujube* Mill.) production requires understanding the specific fertilization needs of jujube trees. This study was conducted to compare fruit yields, phenolic profiles and antioxidant activity of jujube in response to different fertilizers. Application of organic fertilizer appeared to enhance the phenolics and antioxidant activity accumulation of jujubes, compared to conventional fertilized jujubes. Amongst inorganic fertilizers, supplemental potassium as an individual nutrient improved the accumulation of phenolics in jujubes. Our results demonstrate that phenolics levels and antioxidant activity of jujube can be manipulated through fertilizer management and tracked by following proanthocyanidin concentrations. In a practical production context, the combination of organic fertilizers and inorganic fertilizers such as more supplemental individual potassium, and less supplemental individual nitrogen and phosphorus, might be the best management combination for achieving higher phenolic concentration, stronger antioxidant activity and a good harvest.

## 1. Introduction

Jujube (*Ziziphus jujube* Mill.) has been known as a native fruit in China for at least 4,000 years [1]. Nowadays, jujubes have flourished and are widely distributed in Asia, Australia and Europe [2]. This fruit is not only flavorful, but also commonly used in Traditional Chinese Medicine for detoxification, preventing anemia, analeptic, palliative and immunity improvements [1]. Beside the designated uses, jujubes trees are attractive to researchers and producers for their drought tolerance to severe water deficits [3,4].

In China, jujube is being more popular for offering a perennial woody species that reduces soil erosion while producing an economic crop following the government policy of converting small grain production to conservation forestry on the Loess Plateau. For more jujube production, there is an urgent need for the specific fertilization of jujube trees. Traditional intensive agriculture aimed at maximum productivity with large amounts of inorganic fertilizers and pesticides are now being blamed for declining soil fertility and negative environmental effects [5]. The optimal application of appropriate fertilizers for sustainable soil management and economic yield is very important for jujube production. The presence of abundant phenolics in jujube may explain the health-promoting effects observed *in vitro*, *in vivo*, and in nutritional trials with humans [2]. In the effort to improve dietary antioxidant contents, phenolics have often been the target for enhancement. We have previously reported the effects of varieties, ripening stages and processing methods on the accumulation of phenolics in jujube [6,7,8]. Phenolics in plant tissues might also be affected by environmental factors such as fertilizers (nutrient supply) [9]. It has been postulated that relative differences in release of nutrients from various fertilizers could lead to different carbon/nitrogen ratios and consequently differences in the production of secondary metabolites (such as phenolics) in plants [10]. This topic is of high relevance to phenolic production in plants and there is a particular interest in understanding the potential effects of fertilizers. Several previous studies have observed phenolic variation in strawberry, tomato and sea buckthorn berry grown with different fertilizers, but were somewhat inconclusive [11,12,13]. To the best of our knowledge, there is absence of information about the effects of different fertilizers on the phenolics and antioxidant activity of jujube fruit. In the present work, a popular fresh-eaten cultivar of *Ziziphus jujube* Mill., called ‘pear-jujube’ or ‘Lizao’ was selected as the experimental plant. The goal of our study was to evaluate the effect of different fertilizers on the variation in total phenolic content (TPC), total flavonoid content (TFC), total proanthocyanidin content (TPA), antioxidant activity and individual phenolic compounds in jujube (*Ziziphus jujube* Mill. cv. Lizao). Thus, in the present work, organic fertilizer [biogas residue fertilizer (BRF) and decomposed soybean meal fertilizer (DSM)] were compared with inorganic fertilizer [complete NPK fertilizer (NPK)] and an unfertilized control (CK) on the accumulation of phenolics and antioxidant properties in jujube. Moreover, the comparisons of NPK to the individual inorganic nutrients [nitrogen (N), phosphorus (P), potassium (K)] were studied. Furthermore, we investigated the relationship between antioxidant activity and phenolics.

## 2. Results and Discussion

In the present study, jujubes grown with different fertilizers were evaluated and compared for their yields, TPC, TFC, TPA, antioxidant activity and phenolic compounds for the first time. As obtained, jujube was abundant in phenolics and displayed significant antioxidant activity, similar to our previous studies [6,7,8]. Table 1 presents that jujube yields significantly increased with all fertilizer application in respect of CK, which is similar to the results of a previous study in apricot [14]. Complex NPK fertilizer is the most efficient fertilizer in the yields of jujube trees (Table 1), while organic fertilizer is not so efficient. These results are in agreement with previously published information on palm [15] and apricot [14]. Application of individual potassium to jujube trees produces a much higher yield than phosphorus input (Table 1). The beneficial action of potassium has been discovered on the citrus tree [16].

**Table 1:** The experimental fertilizer management and yields of jujube trees.

Plots	Treatment	Fertilizing amount (g/tree)	Yields (kg/tree)
Inorganic Fertilizer	Nitrogenous Fertilizer (N)	545 g (Nitrogen)	11.56 ± 1.26 ^ab^
	Phosphatic Fertilizer (P)	120 g (Phosphorus)	10.51 ± 0.70 ^b^
	Potassic Fertilizer (K)	251 g (Potassium)	11.98 ± 1.15 ^ab^
	Complete NPK Fertilizer (NPK)	220 g (Nitrogen) 120 g (Phosphorus) 251 g (Potassium)	13.22 ± 1.05 ^a^
Organic Fertilizer	Biogas Residue Fertilizer ^©^ (BRF)	5000g (organic matter: 50.65 g/kg FW; N: 30.06 g/kg FW; P: 4.96 g/kg; K: 12.14 g/kg)	10.77 ± 1.19 ^b^
	Decomposed Soybean Meal (DSM)	4000 g ^©^ (organic matter: 76.90 g/kg FW; N:41.31 g/kg FW; P: 9.08 g/kg; K: 15.74 g/kg)	11.21 ± 1.20 ^b^
CK	unfertilized control (CK)	0	8.33 ± 0.43 ^c^

Data expressed as the mean ± SD (n = 3). Means within the same column followed by different letters were significantly different at *p* < 0.05.

**Figure 1:**
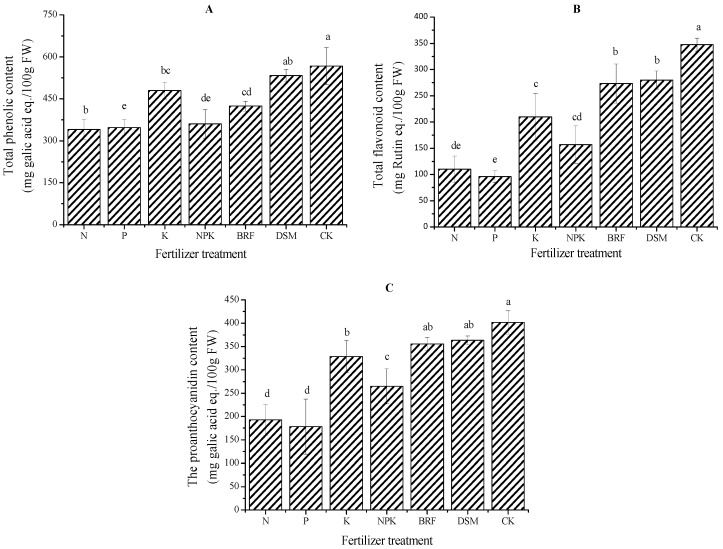
Changes of total phenol, flavonoid and proanthocyanidin content of jujube produced with different fertilizer treatments.

Phenolics, especially the proanthocyanidins in fruits, are of great interest by consumers and researchers because of their potent antioxidant capacity and possible protective effects in human health [11]. TPC, TFC and TPA levels of jujube in response to different fertilizer treatments are presented in Figure 1. Jujubes with fertilizer treatment displayed dramatically lower TPC, TFC and TPA than CK (Figure 1). The CK jujube trees grown in the local natural environment resulted in higher phenolic content in the fruit compared to the fertilization treatments, but growing jujube naturally on the infertile Loess Plateau without fertilization reduces yields substantially. Abundant phenolics in CK jujubes are partially due to the infertile soil induced an unfavorable growing environment, and so that jujube allocated more resources to synthesize defensive phenolics to protect limited nutrient resources from diseases or insects [17,18].

Consumers often perceive that organically grown fruits are of better quality, healthier and more nutritious than conventionally-produced counterparts [19]. When comparing jujubes grown with organic fertilizer (BRF and DSM) to conventional chemical fertilizer (complete NPK), organic fertilizer is efficient in accumulating TPC, TFC and TPA (Figure 1). These results are in agreement with previous observations that higher phenolic concentrations occur in organically fertilized peach and pear [20], marionberry [21], strawberry [22] and tomato [12]. The above result may be partially attributed to the differences in nutrient sources between organic and the complete NPK inorganic fertilizer. The organic fertilizers BRF and DSM have large amounts of carbon-rich organic matter (*i.e.*, humic acid or humus) that could replenish the soil organic matter and improve soil structure and fertility [23,24]. As known, the balance of carbon to nitrogen is necessary to produce phenolics in plants [10]. Due to the plants cannot allocate the resources to growth simultaneously, added organic fertilizer with abundant carbon may push jujube tree to allocate more resources to synthesize phenolics [18,25].

**A**: total phenolic content; **B**: total flavonoid content; **C**: total proanthocyanidin content. Inorganic fertilizer treatments (N, P, K and NPK), organic fertilizer treatments (BRF and DSM) and CK (unfertilized control). The vertical bars represent the SD (n = 3) and bars marked with no letters in common are significantly different (*p* < 0.05).

Inorganic fertilizer is constantly used in fruit production because it is cheap and has high impact on the yields of jujube trees. Supplemental potassium as an individual nutrient improved the accumulation of TPC, TFC and TPA (Figure 1), similar to what was seen in apricot fruit [26]. Such an impact could be partially due to potassium activating physiological metabolism reactions in plants and playing an important role in growth and water-use efficiency [27]. The individual nutrient nitrogen application decreased the TPC, TFC and TPA (Figure 1), consistent with studies of *Arabidopsis thaliana* [28] and broccoli heads [29]. Negative effects of nitrogen on phenolics can possibly be attributed to competition for phenylalanine, which can either be used in phenolic synthesis, or be incorporated into protein synthetic pathways [30,31]. Meanwhile, nitrogen is efficient in improving the protein accumulation [28,29], and so phenolic levels are decreased for a certain amount of phenylalanine. As evaluated in our study, phosphorus showed negative impacts on the accumulation of phenolics in jujube, except for protocatechuic acid. This response is in contrast to findings on garden sage [32], but such inconsistency could be attributed to species differences. Finally, the minimal impact of the complete NPK fertilizer could be due to the synergistic effects of supplemental nitrogen, phosphorus and potassium. The addition of nitrogen and phosphorus may reduce the accumulation of phenolics in jujubes. We believe that more potassium with less phosphorus and nitrogen added are beneficial to the phenolics in jujubes. The detailed interactions among the available nutrients and other environmental conditions will be explored in our future work.

**Figure 2:**
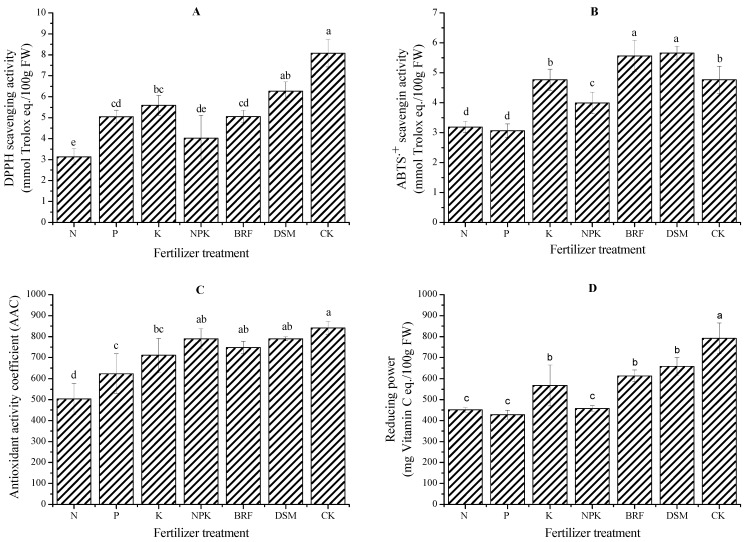
The antioxidant activity of jujube fruit produced by different fertilizers.

A large screening study of fruit extracts from a number of species reported variable concentrations of antioxidant compounds, which sometimes did not correlate with antioxidant activity [33], owing to a mixture of their synergistic and antagonistic interactions. For comprehensive understanding, four assays including electron or radical scavenging and lipid peroxidations were established to evaluate the antioxidant activity induced by jujube trees fertilized by different fertilizers. The pattern of DPPH and reducing power among all these fertilizer treatments somewhat mirrored the above tendency, with the CK having the highest, followed by the two organic treatments and K treatment (Figure 2). However, ABTS^+^ scavenging activity of all treatments (Figure 2) are changed significantly, and the organic fertilizer treatments BRF and DSM are the highest, followed by the CK and K treatments. In addition, the impact of fertilization treatments on antioxidant activity coefficient (AAC) showed the fewest differences and did not follow an existing pattern (Figure 2). As known, the β-carotene-linoleic acid assay determines the lipid peroxidations of antioxidants [34]. Therefore, this pattern of AAC suggested that the phenolics subject to lipid peroxidations in jujubes were not changed obviously following different fertilizer treatments. 

As for individual phenolic compounds, protocatechuic acid, catechin, epicatechin and rutin in jujubes were carefully monitored and their responses to different fertilizers compared (Table 2). As reported in Table 2, the changes in phenolic compounds were complicated and inconsistent. Unexpectedly, only rutin changes follow TPC, TFC and TPA. Organic fertilizer BRF and DSM treatments resulted in higher contents of the four phenolic compounds than complete NPK treatment. Meanwhile, the catechin, epicatechin and rutin contents of K-fertilized jujube were remarkable in the inorganic fertilizer treatments.

Jujubes produced by different fertilizers differ in their antioxidant capacity and phenolic composition. As evaluated in our study, levels of quantified phenolics could not explain all the changes of antioxidant activity with the different fertilizer treatments. This finding was attributed to the synergistic and antagonistic effects from individual phenolics on their expressed antioxidant activity [35,36]. Therefore, the correlations amongst the TFC, TPC, TPA and antioxidant activity were evaluated in this study (Table 3). Interestingly, TPA showed remarkable correlations to TPC, TFC and antioxidant activity. Evaluated in our study, proanthocyanidins might be one of the major phenolic compounds affected by fertilizers. In other words, phenolics and antioxidant activity of jujube can be manipulated through fertilizer management and tracked by following proanthocyanidin concentrations.

**A:** DPPH scavenging activity; **B:** ABTS^+^ scavenging activity; **C:** antioxidant activity coefficient; **D:** reducing power. Inorganic fertilizer treatments (N, P, K and complete NPK), organic fertilizer treatments (BRF and DSM) and CK. The vertical bars represent the SD (n = 3) and bars marked with no letters in common are significantly different (*p* < 0.05).

**Table 2:** Phenolic compounds in the jujube fruit grown by different fertilizers (mg/100g FW).

Treatment	Protocatechuic acid	(+)-Catechin	(−)-Epicatechin	Rutin
N	3.82 ± 0.68 ^c^	22.48 ± 4.79 ^b^	33.18 ± 6.70 ^b^	3.49 ± 0.44 ^d^
P	7.18 ± 1.31 ^b^	28.38 ± 1.76 ^a^	42.62 ± 3.65 ^ab^	3.34 ± 1.17 ^d^
K	4.82 ± 0.67 ^c^	34.11 ± 3.89 ^a^	47.36 ± 8.02 ^a^	6.18 ± 0.24 ^c^
NPK	4.30 ± 0.58 ^c^	29.56 ± 0.43 ^a^	42.66 ± 5.97 ^ab^	3.98 ± 0.72 ^d^
BRF	5.70 ± 0.93 ^bc^	30.97 ± 2.82 ^a^	49.13 ± 7.30 ^a^	5.78 ± 0.51 ^c^
DSM	6.04 ± 0.20 ^bc^	33.68 ± 3.13 ^a^	54.19 ± 2.55 ^a^	8.52 ± 1.17 ^b^
CK	14.08 ± 2.36 ^a^	28.50 ± 3.60 ^a^	43.57 ± 7.31 ^ab^	11.52 ± 1.80 ^a^

Inorganic fertilizer treatment (N, P, K and NPK), organic fertilizer treatment (BRF and DSM) and CK. Data expressed as the mean ± SD (n = 3) and micrograms per 100 gram of fresh weight. Means within the same column followed by different letters were significantly different at *p* < 0.05.

**Table 3:** Correlation analysis among TPC, TFC, TPA, antioxidant activity and phenolic compounds.

	TPC	TFC	TPA	DPPH	ABTS	AAC	Reducing power
TPC	1	0.909 **	0.902 **	0.905 **	0.75	0.711	0.943 **
TFC	0.909 **	1	0.977 **	0.828 *	0.851 *	0.793 *	0.976 **
TPA	0.902 **	0.977 **	1	0.775 *	0.907 **	0.828 *	0.926 **
Protocatechuic acid	0.641	0.637	0.508	0.860 *	0.175	0.51	0.745
(+)-Catechin	0.562	0.483	0.617	0.471	0.738	0.66	0.381
(−)-Epicatechin	0.559	0.569	0.66	0.473	0.822 *	0.751	0.443
Rutin	0.963 **	0.928 **	0.875**	0.925 **	0.654	0.716	0.978 **

** Correlation is significant at the 0.01 level (2-tailed, *p* < 0.05); * Correlation is significant at the 0.05 level (2-tailed, *p* < 0.05). In this Table, TPC: total phenolic content; TFC: total flavonoid content; TPA: total proanthocyanidins content; DPPH: DPPH scavenging capacity; ABTS: ABTS^+^ scavenging activity; AAC: antioxidant activity coefficient in β-carotene-linoleic acid model.

## 3. Experimental

### 3.1. Fruit Tree Orchards

The experimental orchards were located at the Northwest Agriculture and Forestry University Experimental Station in mid-Shaanxi Province on the Loess Plateau (Mizhi County-Yulin, 110°17'E, 37°36'N; average elevation, 1,049 m). The orchards had a Loess soil profile (bulk density: 1.2 g/cm3; available nitrogen: 34.7 mg/kg; available phosphorus: 2.9 mg/kg; available potassium: 101.9 mg/kg; organic matter: 2.1 g/kg; pH: 8.6). Mean annual precipitation for this area was roughly 393 mm, falling largely from July to September. A popular fresh-eaten jujube (*Ziziphus jujube* Mill.) cultivar called ‘pear-jujube’ or ‘Lizao’ was selected in this study. All the experimental dwarfed five-year-old ‘pear-jujube’ trees selected were approximately 2 m high, healthy and uniform, planted by the randomized block design in a 3 m within-row and 2 m between row spacing. Jujube trees were drip irrigated to maintain optimum plant water status. These trees were mainly irrigated at a flow rate of 4 L/h for 4 h in the blooming, fruit setting and developing stages, respectively.

Six fertilizer treatments were randomly applied to ten single-tree replicates per treatment: two organic (BRF and DSM) and four inorganic (complete NPK, individual N, P, K) in addition to the unfertilized control (CK). Fertilizer rates (Table 1) were sufficient and optimal for jujube production in this area according to previous studies and practices over several years. No pesticides were used in our experiments. Fertilizer treatments were repeated for years 2008 and 2009. To avoid potential confounding effects of previous production practices at this location, fruit was analyzed from the second-year’s harvest.

### 3.2. Sample Preparation

Jujube fruit was selected from four sides (east, south, west and north) of each experimental tree. Fruit samples were delivered to the refrigerator immediately after harvest and storage at 0 °C–4 °C for subsequent extraction analyses in two days. The moisture content of the jujube was 80.6%.

Antioxidants in jujube were extracted according to a previously described laboratory procedure [7,8]. Representative homogenized samples (20 g) were extracted with aqueous methanol (60 mL, 80%, v/v) under ultrasonic irradiation for 20 min. Supernatants were collected and residue was repeatedly extracted twice. All the collected supernatants were evaporated at 45 °C, brought to a final volume of 25 mL with absolute methanol, and then stored at −20 °C until further analysis.

### 3.3. Total Phenolic, Flavonoid, Proanthocyanidins Determination

The total phenolic content (TPC) in jujube was assessed using the Folin-Ciocalteu phenol method [7,8]. Results were expressed as gallic acid equivalents in mg per 100 g of fresh weight (mg gallic acid equation/100g FW). The total flavonoid content (TFC) was determined following a modified colorimetric method described by our laboratory [7,8]. TFC was expressed as rutin equivalents in mg per 100 g of fresh weight (mg rutin equation/100g FW). The total proanthocyanidins content (TPA) was determined using the *n*-BuOH/HCl method as modified by our laboratory [7,8]. Results were expressed as grapeseed proanthocyanidins extract (GSPE) equivalents in mg per 100 g of fresh weight (mg GSPE equation/100g FW).

### 3.4. Antioxidant Activity Analysis

DPPH Scavenging Activity. The DPPH free radical scavenging capacity of jujube extracts was evaluated according to a previously described laboratory procedure [7,8]. The extracts with an addition of DPPH reagent solution was left to react in the dark for 30 min. After this duration, the absorbance of the mixture was measured at 517 nm against a methanol blank. The DPPH scavenging activity of antioxidant extracts was expressed as mmol of Trolox equivalent per 100 g of fresh weight (mmol Trolox equation/100g FW).

ABTS^+^ Scavenging Activity. The ABTS^+^ scavenging activity of jujube extracts was evaluated according to a previously reported protocol [7,8]. A mixture of ABTS^+^ solution (5 mM ) and excess manganese dioxide was left to incubate in the dark for 48 h to form the ABTS^+^ radical cation. The mixture was then filtered and diluted with a phosphate buffer solution (0.5 mol/L, pH 7.4) to an absorbance of 0.7 at 734 nm. The final reaction mixture contained ABTS^+^ solution and the extracts. Absorbance of the mixture at 734 nm was determined after 1 min. Results were expressed in terms of mmol Trolox equivalents per 100 g fresh weight (mmol Trolox equation/100g FW).

β-Carotene-linoleic Acid Assay. The antioxidant activity of the extracts was tested in a β-carotene-linoleic acid system using a previously described method [7,8]. β-Carotene (2 mg), linoleic acid (45 mg) and Tween-40 (350 mg) were dissolved and made up to a volume of 10 mL with chloroform. The chloroform was then removed through the rotatory evaporation, and oxygen-saturated distilled water was added to form an emulsion. Extracts were blended with the emulsion and incubated for 60 min at 50 °C. Absorbance was read at 470 nm using a spectrophotometer against a blank (emulsion with no β-carotene) at 0 min and 60 min. Antioxidant activity measured in the β-carotene-linoleic acid assay was expressed as antioxidant activity coefficient (AAC) calculated using the following equation:

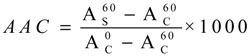

where 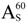 is the absorbance of the sample at 60 min, 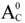 is the absorbance of the control sample at 0 min and 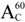 is the absorbance of the control sample at 60 min.

Reducing Power Determination. The reducing power model was established according to a previously reported version [7,8]. Jujube extract was mixed with phosphate buffer (0.2 mol/L, pH 6.6) and potassium ferricyanide (1%, w/v) and incubated at 50 °C for 20 min. Afterward, trichloroacetic acid (10%, w/v) was added to the mixture to stop the reaction. A portion (1 mL) of the reaction mixture was then blended with distilled water and ferric trichloride solution (0.1%, w/v). The resulting mixture was incubated in the dark for 30 min, and absorbance was measured at 700 nm. Results were expressed in terms of vitamin C equivalents in milligrams per 100 g fresh weight (mg vitamin C equation/100g FW).

### 3.5. Phenolic Compounds Analysis

Phenolic compounds were evaluated using a previously reported laboratory procedure with slight modifications [7,8]. Fresh jujube fruit (20 g) from each treatment was extracted with ethyl acetate (AR, 40 mL). The mixture was extracted in water under ultrasonic irradiation for 30 min and centrifuged at 4,000 rpm for 10 min. Supernatant was collected and the residue was re-extracted twice. All the collected supernatants were evaporated to dryness at 35 °C. The residue was then brought to 5 mL volume with absolute methanol. The extracts were stored in the dark at −20 °C and subsequently analyzed by high-performance liquid chromatography (HPLC).

HPLC analysis was performed on a LC-2010AHT chromatograph instrument equipped with a UV-vis detector (Shimadzu Corp., Kyoto, Japan). Samples were injected at ambient temperature (30 °C) into a reverse phase Waters symmetry C18 column (4.6 mm × 150 mm, particle size 5 µm) with a gradient of solvent A (100% methanol) and solvent B (pH 2.6: ultrapure water acidified with phosphate) at a flow rate of 0.8 mL/min. The solvent gradient was programmed as follows: at 0 min, 15% A; at 15–25 min, 25% A; at 65 min, 75% A; at 70 min, 15% A. Phenolic compounds in the eluents were monitored at 280 nm. Seven phenolic compounds, gallic acid; protocatechuic acid; (+)-catechin; chlorogenic acid; caffeic acid; (−)-epicatechin; rutin and cinnamic acid, were supposed to be present in jujube [37,38]. Identification of the phenolic compounds was accomplished by comparing the retention times and spectra in samples to those of phenolic compounds standards. Each sample was analyzed three times by HPLC.

### 3.6. Statistical Analysis

Data from this study were expressed as mean ± standard deviations (SD) for the single-tree replicate determinations of each fertilizer treatment. Variance among data was compared by Duncan’s test, and a Pearson correlation test between means was analyzed by PASW Statistics 18 (SPSS Inc., Chicago, IL, USA). Statistical significance was declared at *p*<0.05. 

## 4. Conclusions

Jujube fruit produced by different fertilizers differ in their antioxidant capacity and phenolic composition. Natural growing practices induce high phenolic concentration and strong antioxidant activity at the cost of much lower yields. Organic fertilizers and inorganic fertilizers such as more potassium, less nitrogen and phosphorus are efficient in simultaneously improving yields, phenolics levels and antioxidant activity. Proanthocyanidins could be the major compounds among the phenolics affected by fertilizers and were used as a proxy to estimate the effects of fertilizers on the phenolic concentration and antioxidant activity. These results clearly demonstrate that phenolics and antioxidant activity in jujubes depend on fertilizer type and could be tracked by following proanthocyanidin concentrations. In orchards, phenolics and antioxidant activity of jujubes could be manipulated through fertilizer management.
